# Low bone mineral density following gastric bypass is not explained by lifestyle and lack of exercise

**DOI:** 10.1186/s12893-021-01281-5

**Published:** 2021-06-04

**Authors:** Katharina Stevens, Hella Hultin, Per Hellman, Magnus Sundbom

**Affiliations:** Department of Surgical Sciences, Uppsala University, Entrance 70, University Hospital, 751 85 Uppsala, Sweden

**Keywords:** Bone mineral density, Gastric bypass, Vitamin D, Lifestyle, Long-term results

## Abstract

**Background:**

Bariatric surgery, Roux-en-Y gastric bypass (RYGBP) in particular, is associated with weight loss as well as low bone mineral density. Bone mineral density relies upon multiple factors, some of which are lifestyle factors. The aim of this study was to compare lifestyle factors in order to eliminate them as culprits of the suspected difference in BMD in RYGBP operated and controls.

**Materials and methods:**

Study participants included 71 RYGBP-operated women (42.3 years, BMI 33.1 kg/m^2^) and 94 controls (32.4 years, BMI 23.9 kg/m^2^). Each completed a DEXA scan, as well as survey of lifestyle factors (e.g. physical activity in daily life, corticosteroid use, and calcium intake). All study participants were premenopausal Caucasian women living in the same area. Blood samples were taken in RYGBP-patients.

**Results:**

BMD was significantly lower in RYGBP, femoral neck 0.98 vs. 1.04 g/cm^2^ compared to controls, despite higher BMI (present and at 20 years of age) and similar physical activity and calcium intake. In a multivariate analysis, increased time since surgery and age were negatively associated with BMD of the femoral neck and total hip in RYGBP patients.

**Conclusion:**

Despite similar lifestyle, RYGBP was followed by a lower BMD compared to controls. Thus, the reduced BMD in RYGBP cannot be explained, seemingly nor prevented, by lifestyle factors. As the reduction in BMD was associated with time since surgery, strict follow-up is a lifelong necessity after bariatric surgery, and especially important in younger bariatric patients.

## Background

An increasing amount of studies have shown reduced bone mineral density in patients after bariatric surgery, with the surgical procedure being a suspected cause; however few studies to our knowledge have considered the impact lifestyle factors may have upon bone mineral density in this group.

At present, bariatric surgery is the most effective treatment of obesity. The number of surgical bariatric procedures performed worldwide has continued to increase each year with almost 700,000 procedures reported during 2016 [1]. Roux-en-Y gastric bypass (RYGBP) has had a long tradition in the United States, averaging approximately 113,000 surgeries per year at a cost of approximately $1.5 billion annually [2, 3]. In Sweden, bariatric surgery has increased ten-fold since 2002, and RYGBP remains one of the most common bariatric procedures performed [4].

RYGBP has several working mechanisms; reduced size of the stomach, excluded passage through the duodenum and proximal small bowel, and multiple described changes in gastrointestinal hormones [5]. Overall, the changes in the physiology of the gastrointestinal tract result in significant weight loss (average roughly 15 BMI-units), in addition to an increased risk of a variety of malnourishment conditions including vitamin D and calcium deficiency. Studies have found that after bariatric surgery 10–25% of patients develop a calcium deficiency by 2 years and 25–48% by 4 years [6]. In individuals with low calcium intake, absorption relies upon vitamin D metabolites that increase the number of calcium channels in the duodenum. Therefore, when the duodenum is bypassed after RYGBP, a patient with low calcium intake is not able to compensate via vitamin D dependent mechanisms. This may lead to metabolic bone disease, where the building of normal bone strength is compromised due to malnourishment. In addition, to maintain circulating levels of calcium bone may be resorbed, which is evident in upwards to 30% of RYGBP patients post-operatively [7].

The integrity of the skeletal system can be evaluated by measuring bone mineral density (BMD). Throughout the first three to four decades of life BMD increases to peak levels before beginning a slow decline. Peak BMD levels rely upon adequate nutrition and physical activity as well as limited exposure to detrimental factors such as smoking and certain medications. It is widely accepted that higher peak BMD achieved in early adulthood is protective against low BMD later in life [8]. As the age of patients undergoing bariatric surgery continues to creep lower, we suspect this to risk reduced peak BMD in this group.

Low bone mineral density is a well-known risk factor for fracture and is therefore associated with increased risk for morbidity. The global burden of low BMD has grown steadily over the past decades, which is likely attributable to an aging population [9]. A DEXA scan measures the BMD of a patient, which is then compared to standardized measurements obtained from healthy populations matched for sex. Low scores obtained from a BMD scan may lead to a diagnosis of osteopenia or osteoporosis. Threats to BMD also include prolonged corticosteroid use, alcohol consumption, smoking, and increased age [10, 11]. Bone matter adapts to increased strain by increasing in density, and the opposite is also true of decreased bone mineral density [12]. Therefore, obesity and physical activity have been cited as protective factors against osteopenia and osteoporosis whereas weight loss and sedentary behavior have been implicated in decreased BMD measurements [13]. Sedentary behavior is commonly found in studies of bariatric patients, implying that lack of physical activity may be a contributing cause for low BMD in this population [14]. However, several studies have also found that physical activity increases postoperatively in this group, which should theoretically inhibit BMD reduction [15]. Essentially, significant decreases in BMD post-bariatric surgery is an established threat that applies to a growing number of patients; however the cause and prevention of BMD reduction has yet to be properly explored.

The aim of the present study was to compare lifestyle factors in two groups of premenopausal women, RYGBP-operated and controls, to eliminate these as culprits of the suspected difference in BMD. A secondary aim was to evaluate prognostic factors for low BMD in the RYGBP-group.

## Materials and methods

RYGBP-operated patients were contacted through a local registry of RYGBP procedures performed between 1996 and 2006 at Uppsala University Hospital, Sweden. Of the 208 premenopausal Caucasian women of Swedish origin that were contacted, 90 agreed to undergo bone mineral density (DEXA) scanning and complete a survey about her lifestyle factors. The control group consisted of women 20–40 years of age that had not undergone bariatric surgery, living in the Uppsala area. The data was collected by Ribom et al. during a separate study of BMD among ethnically Swedish women [16]. From this initial group of 335 women, 300 women had a BMI greater than 20 kg/m^2^. After excluding women that did not satisfy the criteria of being premenopausal, Caucasian, of Swedish origin and not having undergone bariatric surgery, 149 women took part in the lifestyle survey. Current smokers were excluded from this study to eliminate this as a confounding variable upon BMD measurements, leaving 71 participants in the RYGBP group and 94 in the control group.

Bone mineral density (g/cm^2^) of the femoral neck, total hip, and lumbar spine (vertebrae L1–L4) was measured by DEXA (Lunar Prodigy, Lunar corp., Madison, WI, USA). When applicable, both extremities were used in the calculation. By triple measurements in 15 subjects, the precision error of the DEXA measurements in our laboratory has been calculated to be between 0.8 and 1.5% for BMD depending on site. Daily scans of a lumbar spine phantom were performed and the long-term precision error CV% was less than 1% during the study period. For the bariatric group Lunar Prodigy was used, whereas the control group was measured using Lunar DPX-IQ. To compare the measurements from the two DEXA densitometers a cross-calibrations was performed, DPX_BMD_ = 0.08 + 0.906*Prodigy_BMD_ [17].

The lifestyle survey included health related events (smoking, fractures, menarche, and current medications), approximations of calcium intake from milk and cheese by direct questions on consumption of cheese and milk, average time spent weekly on various types of physical activity, e.g. jogging, walking, trekking and aerobics (present and a 5-year approximation), as well as current weight. The average calcium contents in each slice of cheese and deciliter of milk were calculated by using information listed by the Swedish Food Agency. Physical activity was measured by estimating minutes per week spent on commonly performed moderate-to-vigorous physical activities by adults in Sweden [18]. RYGBP-patients were also asked the duration of their obesity, preoperative weight, and date of surgery.

Statistics were analyzed using SPSS Statistics software (version 17.0; SPSS, Chicago, IL, USA). Descriptive statistics are presented as mean and SD, or number of patients and percent. Normality was tested for using Shapiro-Wilks test. Comparisons between groups were done by Students t-test for parametric data, non-parametric continuous data was analyzed using Mann–Whitney test, and χ^2^-test for categorical data, with p < 0.05 considered statistically significant. The effect of the studied variables on BMD was analyzed by linear regression, where all variables with p < 0.1 in univariate analysis were entered into a multivariate model. For this analysis, the control group was included as having “0” as postoperative time, whereas RYGBP patients had their corresponding value. Prognostic factors for low BMD in the RYGBP-group were studied in an identical manner.

This study was conducted in accordance to the 1964 Declaration of Helsinki and subsequent amendments as well as the ethical standards of the institution.

## Results

### Lifestyle survey

Age and current BMI differed between groups, as demonstrated in Table 1. No difference was seen in calcium intake nor in physical activity reported at present and 5 years ago. No significant difference in corticosteroid use, known to decrease BMD, was seen either*.*

**Table 1:** Demographic and lifestyle information for both groups in addition to bone mineral density (BMD) measurements

Variable	RYGBP (n = 71)	Controls (n = 94)	p-value
Age (years)	42.4 ± 5.6	32.4 ± 5.0	< 0.001
BMI (kg/m^2^)	33.0 ± 5.8	23.9 ± 2.9	< 0.001
Corticosteroid use (yes/no)	9/62	7/66	0.556
Menarche (years)	12.56	12.63	0.825
Calcium intake (mg/day)	681 ± 397	327 ± 126	< 0.001
Activity, 5 years ago (min/week)	208 ± 248	287 ± 336	0.146
Present activity (min/week)	312 ± 356	315 ± 306	0.886
BMD femoral neck	0.98 ± 0.12	1.04 ± 0.13	0.004
BMD hip	1.02 ± 0.12	1.07 ± 0.13	0.008
BMD spine	1.22 ± 0.13	1.24 ± 0.14	0.487

Means ± standard deviations of continuous variables in each group compared using student’s t-test for parametric data, Mann–Whitney for non-parametric, and χ^2^-test for categorical data

### BMD measurements

**Fig. 1:**
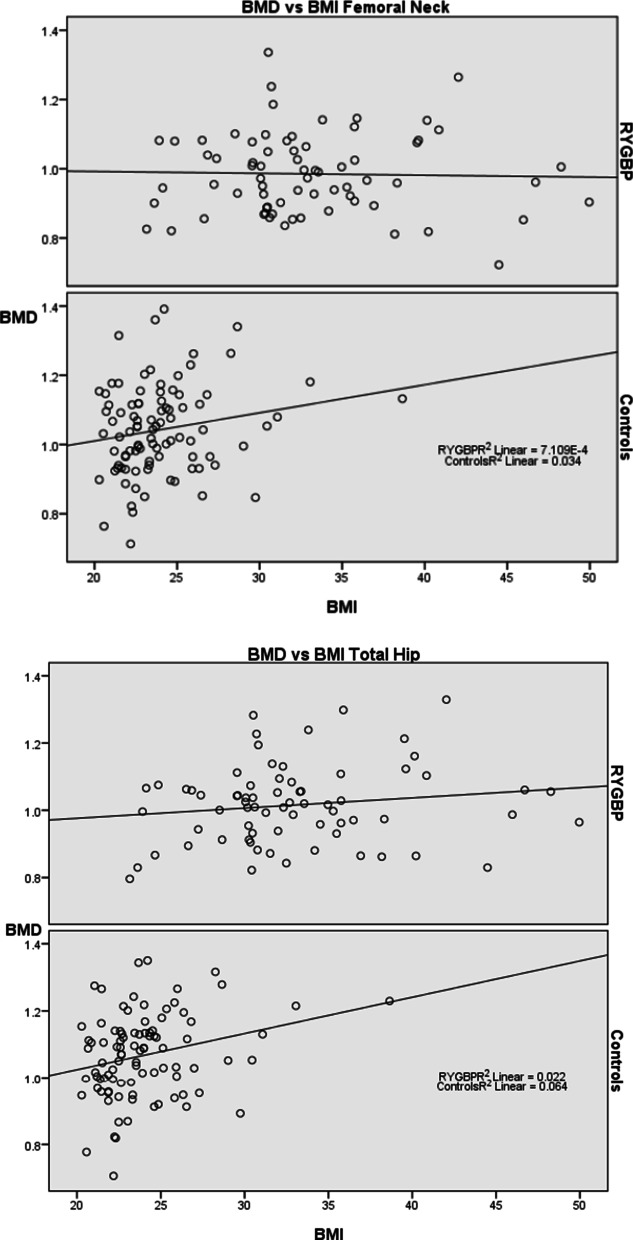
Scatterplot of BMD (g/cm^2^) and BMI (kg/m^2^)

BMD measurements were obtained for all women. BMD of the femoral neck and total hip was significantly lower in RYGBP-patients than controls, 0.98 vs. 1.04 g/cm^2^ and 1.02 vs 1.07 g/cm^2^ respectively. No difference was seen in BMD Spine (1.22 vs. 1.24 g/cm^2^; Table 1). The overall relationship between BMD and BMI in controls and RYGBP-patients is demonstrated in Fig. 1. Figure 1 also illustrates the significant correlation between BMI and BMD of the total hip in the control group, which is not seen in the RYGBP group (r = 0.252, p = 0.015 vs. r = 0.149, p = 0.214).

### Prognostic factors in RYGBP-patients

The RYGBP group achieved a mean weight loss of 34.4 kg (from BMI 45.4 to 33.1 kg/m^2^). The mean age at surgery was 34.7 years, and mean duration of obesity was 23.1 years according to survey results. In a univariate regression analysis, younger age and not having undergone RYGBP were associated with higher BMD of both the femoral neck and total hip. No associations were seen for lifestyle factors. In multivariate analyses, a trend towards association between higher BMD of the femoral neck and younger age (p = 0.09) as well as time since surgery (p = 0.06) was seen. For BMD of the total hip, each postoperative year was associated with a reduction of BMD by 0.005 g/cm^2^, p < 0.05 (Tables 2 and 3).

**Table 2:** Regression analysis with Femoral Neck BMD as dependent variable

Variable	Univariate			Multivariate		
	Coefficient	Standard error	p-value	Coefficient	Standard error	p-value
Age at present	− 0.005	0.001	< 0.001	− 0.003	0.002	0.094
Years since surgery	− 0.008	0.000	< 0.001	− 0.005	0.000	0.062
BMI	− 0.002	0.002	0.118			
BMI at 20 years of age	0.000	0.002	0.773			
Calcium intake	− 0.000	0.000	0.765			
Activity at present (min/week)	− 0.000	0.000	0.705			
Activity 5 years ago (min/week)	0.000	0.000	0.555

**Table 3:** Regression analysis with total hip BMD as dependent variable

Variable	Univariate			Multivariate		
	Coefficient	Standard error	p-value	Coefficient	Standard error	p-value
Age at present	− 0.004	0.001	0.006	− 0.001	0.002	0.412
Years since surgery	− 0.0076	0.000	0.001	− 0.003	0.000	0.026
BMI	0.000	0.002	0.738			
BMI at 20 years of age	0.000	0.002	0.705			
Calcium intake	0.0000	0.000	0.915			
Activity at present (min/week)	− 0.0000	0.000	0.844			
Activity 5 years ago (min/week)	0.0000	0.000	0.711			

The following laboratory values were seen in the RYGBP group, Vitamin D 52.7 mmol/L, intact PTH 8.25 pmol/L, and albumin 36.8 g/L. A trend towards association between BMI at surgery and BMD of the total hip was seen (p = 0.083) (Table 4).

**Table 4:** Prognostic factors for RYGBP patients in univariate analyses with BMD of the femoral neck and total hip as dependent variables

Variable	Univariate BMD femoral neck			Univariate BMD total hip		
	Coefficient	Standard error	p-value	Coefficient	Standard error	p-value
Age at surgery	0.000	0.003	0.862	0.001	0.002	0.620
BMI at surgery	0.002	0.002	0.287	0.004	0.002	0.083
Change in BMI	0.003	0.002	0.162	0.002	0.003	0.538
Vit D (nmol/L)	0.001	0.001	0.252	0.001	0.001	0.460
PTH intact	− 0.003	0.001	0.443	− 0.003	0.003	0.401

## Discussion

As expected, the RYGBP group had significantly lower bone mineral density (BMD) compared to controls. As no significant associations with lifestyle factors (physical activity, BMI, calcium intake and medications) were found, we consider the low BMD to be procedure related.

### BMD related to age

Bone mineral density increases at a varying rate up until roughly the third decade of life where peak bone mineral density is reached and a slow decline in BMD begins [19]. The average age of each group (controls: 32 years; RYGBP: 42 years) places the studied groups roughly at peak BMD levels. All studied individuals were premenopausal, unfortunately the two groups were not identical in age. A larger group of volunteers to select appropriate controls may have solved this, however the impact of age when considering the BMD in premenopausal women may be considered minute. A cross-sectional study over two years of premenopausal adult women found decreased DEXA measurements only of the hip (< 0.003 g/cm^2^/year) whereas other sites did not show significantly decreased BMD measurements until menopause [20]. Therefore, the significant reduction in BMD cannot be explained by age alone in the RYGBP group.

### BMD related to BMI and physical activity

Overall, BMD was positively correlated to BMI. This suspected correlation is in line with the theory of mechanostat that states that bone matter increases in density in response to increased mechanical loading [21]. The mechanostat theory postulates that increased skeletal strain leads to a release of anabolic factors and thereby bone remodeling [22]. Mechanical load increases with BMI and/or physical activity depending upon lifestyle, therefore obesity has been cited as a promoter of high BMD. Both groups reported physical activity of similar amounts. Studied individuals also reported estimates of activity levels at five years prior, giving insight into lifestyle and mechanical loading over time during which peak BMD was established. This is important as higher BMD is seen in individuals that have had a more physically active lifestyle during adolescence and young adulthood, even if physical activity is ceased during adulthood, compared to steady regularly physically active adults [23]. In the control group a positive linear relationship between BMI and BMD was found, however this is not demonstrated as clearly in the RYGBP group (Fig. 1). Our interpretation is that the procedure in itself halts the mechanostat mechanism of bone remodeling, possibly due to malabsorption of skeletal building blocks from the intestine.

### BMD related to calcium intake

Calcium, the building block of the skeleton, is a required nutrient for skeletal growth and bone remodeling. Although found in a variety of dairy and plant-based products, some primary sources of calcium in the Swedish population are milk and cheese. Guidelines for daily intake in Sweden include 800 mg of calcium daily [24]. The Swedish nutritional supplement recommendation for RYGBP patients postoperatively includes 500 mg of calcium and 800 IU of vitamin D [25]. As all patients in the RYGBP group have undergone surgery at the same surgical center, the RYGBP group has had identical post-operative follow-up recommendations. While weight loss in itself, as stated above, leads to a reduction in BMD a study showed that weight loss with a sustained macronutrient diet does not reduce bone mineral density [26]. Interestingly, the American Association of Family Practitioners has listed gastric bypass as a risk factor for developing osteopenia and/or osteoporosis regardless of achieved weight loss. This suggests that deficiency of essential nutrients is a key cause to BMD reductions among bariatric patients. Considering a significantly higher intake of calcium and identical post-surgery supplement recommendations, diet is not identified as a key cause to the lower BMD seen in the RYGBP group.

### BMD related to RYGBP

A link between bariatric surgery and decreased bone density has been suggested in various studies. The mechanism of this, in theory, is the reduced uptake of essential nutrients as mentioned above. In line with the results of this study, the femoral neck and hip have repeatedly been described as sites that have reduced BMD measurements in bariatric patients [27, 28]. Casagrande et al. published evidence of decreased BMD in patients one year after surgery [29]. Another study conducted in 2009 monitored postmenopausal women five years after surgery and found similar trends of decreased BMD, which they concluded was unrelated to vitamin D and calcium intake [30]. The present RYGBP-group had lower BMD than controls despite reporting lifestyle factors that implicate overall greater mechanical stress upon the skeleton over time. The studied RYGBP group underwent identical postoperative follow-up, including monitoring of PTH levels as an indirect sign of calcium deficiency. Interestingly a recent study has shown that PTH levels within normal reference intervals does not conclude adequate nutrition to achieve high peak BMD levels in adolescents. The study concluded that vitamin D was a major determinant of peak BMD, and aberrations in PTH levels were not correlated to peak BMD measurements [31]. Another study found that adherence to a multivitamin regimen over time decreases with time post bariatric surgery, primarily in younger patients which are more dependent upon adequate nutritional status to reach peak BMD [32]. From this we conclude that bariatric patients are at risk for reduced BMD, and that younger patients undergoing surgery may be especially vulnerable if peak BMD is stunted.

### BMD related to medications

Several medications have shown to effect BMD. In accordance to the FRAX tool, a well-established algorithm for 10-year osteoporotic fracture risk, corticosteroids was the only type of medication included in our analysis [33]. Type of therapy, duration of therapy, and dosage were not taken into consideration because of the complexity of the matter, and that the purpose was essentially to provide an illustration of prevalence of potentially confounding variables between groups. Overall, inhaled corticosteroids were the most common type of therapy in the subjects studied, which is considered to have a less pronounced effect upon BMD than daily oral corticosteroid use. Regardless, prevalence of corticosteroid use did not differ between the groups, and therefore we conclude that the low BMD in the RYGBP group cannot be attributed to this medication.

This study has limitations. The study could have been underpowered for individual lifestyle assessment; however, we believe that differences with clinical importance would be found in the present 165 patients. The cross-sectional design does not illustrate causality of the procedure and low BMD. The design also allows for recall bias, especially in lifestyle factors five years ago. Furthermore, the RYGBP group are likely to have more regular diet and lifestyle focused healthcare appointments as part of the routine follow-up after bariatric surgery, which may be reflected in their reported physical activity. Worth noting, ideally the study would have had the same type of DEXA densitometer for each group; however, since both machines were from the same supplier, the measurements have been compared using an established conversion formula, as previously stated [17]. Concerning calcium intake, only milk and cheese consumption were included in the survey as these are two of the most common calcium sources in a normal Scandinavian diet. These items were used as indicators of calcium intake, rather than calculating total calcium consumption. With that stated, vitamin and nutritional supplementation were not accounted for, neither was compliance to supplement regimes. All RYGBP patients are prescribed multivitamins including calcium postoperatively, however it is known that compliance decreases significantly to these supplements over time. With these factors considered and the results of the study, we believe that nutritional calcium and prescription of dietary supplements alone are not enough to prevent low bone mineral density in bariatric patients postoperatively.

The skeletal effects of bariatric surgery are presumably multifactorial, including nutritional factors, mechanical unloading, hormonal factors, and changes in body composition. Lifestyle modifications after bariatric surgery are important to maintain weight loss and to mitigate the adverse impact upon bones. Ultimately, reduced BMD poses an increased risk for fractures. Studies have shown that RYGBP is associated with an increased risk of fracture at osteoporotic sites, a risk that starts to manifest between 2 and 5 years after surgery [34]. In response to these findings the joint guidelines released by the American Association of Clinical Endocrinologists (AACE), the Obesity Society, and American Society for Metabolic and Bariatric Surgery (ASMBS) state that DEXA bone density scans are indicated both preoperatively and 2 years after bariatric surgery [35]. These guidelines have yet to be adopted in Sweden.

As suspected, RYGBP was associated with lower BMD measurements. This study supports the theory that BMD reduction is procedure related as the RYGBP group had lower BMD than controls despite reporting higher skeletal strain and indicating a more calcium rich diet. Further, BMD measurements were significantly inversely associated with postoperative time. From this study we conclude that current postoperative follow-up regimes need to be expanded with a more aggressive approach to screen for and prevent decreases in BMD, and essentially the development of osteopenia/osteoporosis.

## Data Availability

All collective data is present in the manuscript. The datasets generated and/or analyzed during the current study are not publicly available due to patient privacy but are available from the corresponding author upon reasonable request.
